# Rescuing inflammatory microenvironment induced senescence via MgO immune mediated asymmetric short fibers for accelerating diabetic wound repair

**DOI:** 10.1016/j.mtbio.2025.102079

**Published:** 2025-07-13

**Authors:** Yedan Chen, Qingxiang Liu, Jingjing Guan, Chunyang Zheng, Shumeng Shi, Weiwei Zheng, Jianzhong Guan, Yingji Mao

**Affiliations:** a Department of Orthopedics, Department of Plastic Surgery, and Department of Rehabilitation Medicine, The First Affiliated Hospital of Bengbu Medical University, Bengbu, 233004, China; b Anhui Nerve Regeneration Technology and Medical New Materials Engineering Research Center, School of Life Sciences, Bengbu Medical University, Bengbu, 233030, China; c Anhui Provincial Key Laboratory of Tumor Evolution and Intelligent Diagnosis and Treatment, Bengbu Medical University, Bengbu, 233030, China; d Department of Orthopedics, The Affiliated Suzhou Hospital of Nanjing Medical University, Gusu School, Nanjing Medical University, Suzhou, 215000, China

**Keywords:** Metal immunotherapy, Cellular senescence, Electrospinning, Aerogel, Diabetic wound healing

## Abstract

Ongoing disturbance of the immune microenvironment under diabetes mellitus is a significant obstacle to optimal wound repair. Persistence of the local inflammatory niche from incomplete or dysregulated regression of acute inflammation causes the surrounding tissues to undergo immune-induced senescence. Inspired by the natural tissue structure of the extracellular matrix and metal immunotherapy, we developed an asymmetric short-fiber Gelatin/Poly(L-lactic acid) aerogel scaffold loaded with magnesium oxide nanoparticles (GP@MgO aerogel). This innovative short-fiber aerogel scaffold addresses the two-dimensional longitudinal collapse of electrospinning. It achieves a spatial three-dimensional bionic structure that provides additional cell adhesion sites and slowly absorbs tissue exudates. The gravity-induced short fibers form asymmetric densities at both ends, facilitating exudate management. Meanwhile, this aerogel relieves over-activated inflammation and alleviates defects in the cellular energy supply, accelerating neovascularization and reducing the aging process of tissues adjacent to the trauma. Furthermore, GP@MgO modulates macrophage polarization and decreases the release of senescence-associated secretory phenotypes, achieving accelerated revascularization and re-epithelialization in a full-thickness skin model of diabetic rats. In conclusion, these findings indicate that short-fiber GP@MgO aerogels offer a measure to regulate biochemical functions and physiological responses for the rapid closure of diabetic wounds through metal immunotherapy.

## Highlights

• The asymmetric short-fiber aerogel (GP@MgO) was developed.
• GP@MgO aerogel improved the immune microenvironment and down-regulated cellular senescence phenotype.
• GP@MgO aerogel blocked the vicious cycle between inflammation-senescence and rescued inflammaging.
• GP@MgO aerogel accelerated wound closure in diabetic rats.

## Graphical abstract

**Figure:**
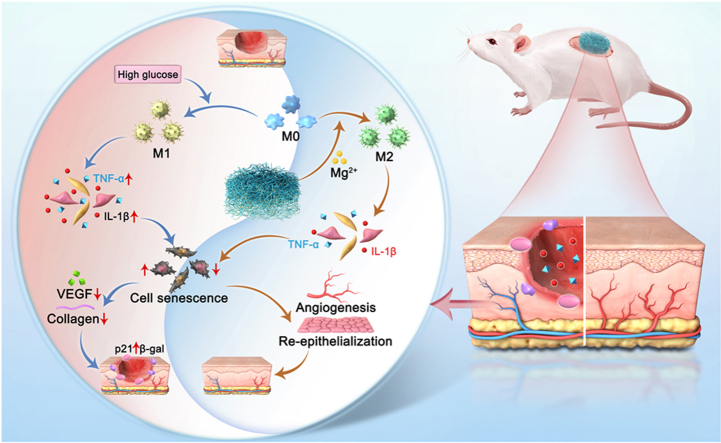

## 1 Introduction

As the body's primary line of physical defense system and largest immune organ, the skin is susceptible to damage caused by external trauma and internal diseases [1,2]. With the rising prevalence of diabetes mellitus, effective wound management is becoming increasingly urgent, particularly since the incidence of diabetic trauma resulting from elevated glucose levels and sustained chronic inflammation is also increasing [3]. Elevated oxidative stress caused by a high-glucose microenvironment can harm the body. This further intensifies localized inflammation, prolongs the inflammatory phase of wound healing, and accelerates inflammatory aging in the host tissue [4,5]. Therefore, to facilitate the regeneration of skin wounds in the complex microenvironment of diabetes mellitus, there is growing interest in using tissue materials to intervene in the physiological processes governing wound regeneration.

The wound-healing process is divided into hemostasis, inflammation, hyperplasia, and remodeling [6]. To achieve optimal wound healing, it is essential to consider the interaction between the complicated diabetes mellitus microenvironment and the host's physiological activities to reduce the time required. Innate immunity represents the initial response following injury, driving neutrophil action and macrophage recruitment [7]. However, the prolonged local presence of M1 macrophages and delayed expression of M2 macrophages are due to high levels of oxidative stress and an abnormally active energy metabolism bypass in diabetes mellitus. The consequence of this macrophage polarization is stagnation of the wound in the inflammatory phase, which can lead to the formation of chronic wounds. Reasonable control of macrophage phenotypic shifts is key to facilitating accelerated processes [8]. Furthermore, mitochondrial function is compromised by several external factors, resulting in reduced membrane potential and impaired ATP production [9,10]. Excessive accumulation of reactive oxygen species (ROS) and continuous secretion of senescence-associated secretory phenotypes (SASP) result in the premature onset of cellular senescence, leading to a vicious cycle. The accumulation of senescent cells also impedes the healing of diabetic wounds [11]. In response to the challenging microenvironment of wounds, there has been significant focus on metal-based materials [12,13].

Metal ions are involved in molecular interactions within the immune system through their structural, catalytic, or regulatory components and represent a promising basis for novel metal immunotherapies [14,15]. For example, metal ions can play a role in cellular signaling by acting as cofactors for enzymes, which can lead to the activation or enhancement of intrinsic and adaptive immunity. Meanwhile, metal ions also influence the expression of inflammatory factors and regulate microbiota composition and growth [16]. This could help alleviate the growing crisis of antibiotic abuse. To illustrate, MgO nanoparticles can competitively inhibit inward calcium flow by altering the local pH environment to inhibit cellular senescence and restore cellular activity [17]. Therefore, metal nanoparticles have attracted considerable attention in tissue engineering as significant components of trace elements in the human body. However, because of potential biotoxicity caused by a rapid increase in the number of ions in the short period directly after use, it is crucial to choose a suitable delivery system to control the release rate, such as decellularized matrices [18], hydrogels [19], microspheres [20], and electrospinning [21].

Electrospinning can be used to arrange micro/nanoscale fiber filaments to mimic the structure of a natural extracellular matrix (ECM) [22,23]. Providing mechanical support and ensuring biological survival by selecting suitable raw materials are crucial for optimal bionic performance. Poly (L-lactic acid) (PLLA) was incorporated to balance the hydrophilicity of pure gelatin with the lack of mechanical properties inherent in this material [24]. Meanwhile, the high specific surface area of electrospinning fibers provides additional attachment points for cell adhesion and proliferation, enabling it to play a role in the coordinated evolution of intrinsic cells and precise structural remodeling of the microenvironment [25]. However, conventional fibrous membranes are two-dimensional structures formed by dense fiber stacks with small pore sizes; the lack of three-dimensional structures presents a challenge for their practical application [26]. Therefore, short-fiber aerogel scaffolds with high porosity and three-dimensional stereoscopic structures were constructed by remodeling the fiber structure after crushing. Owing to its unique morphology and microporous structure, the scaffold guarantees material and nutrient exchange between cell growth and tissue remodeling [27].

**Scheme 1:**
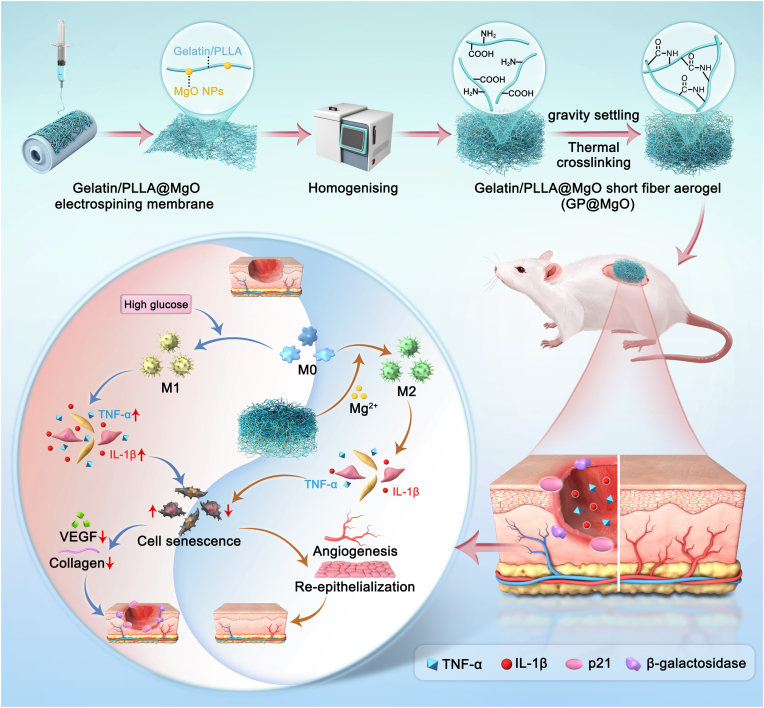
Schematic illustration of asymmetric GP@MgO short fiber aerogel and therapeutic measures for rescuing immune-induced senescence and accelerating wound closure in diabetic rats.

Herein, inspired by the natural tissue structure of the ECM and the biochemical properties of metal ions, MgO NPs were added to a PLLA/Gelatin solution, and porous short-fiber aerogel scaffolds were prepared through electrospinning, pulverization, freeze-drying, and cross-linking (Scheme 1). The Gelatin/PLLA@MgO (GP@MgO) aerogel with a three-dimensional short-fiber structure was designed to slowly absorb wound exudate while releasing loaded MgO NPs in a gradual and sustained manner. We demonstrated that GP@MgO is biocompatible and effective at inhibiting inflammatory responses and slowing cellular senescence. Furthermore, GP@MgO significantly accelerated revascularization and re-epithelialization, promoting wound repair in diabetic rat skin defects. In conclusion, GP@MgO short-fiber aerogel scaffolds provide a measure to regulate biochemical functions and physiological responses in the context of wound healing in a complex microenvironment.

## 2 Materials and methods

### 2.1 Materials

PLLA was purchased from Jinan Daigang Co. (Jinan, China). Gelatin (Gel) was purchased from Sigma-Aldrich (Shanghai, China). The 1,1,1,3,3,3-Hexafluoro-2-propanol (HFIP) and tert-butanol were purchased from Shanghai Aladdin Co. (Shanghai, China). Cell culture reagents were obtained from Gibco (Carlsbad, CA, USA).

### 2.2 Fabrication of GP@MgO aerogel

MgO NPs were synthesized as previously described [28]. The aerogel was fabricated using a previously reported technology that combines electrospinning, pulverization, freeze-drying, and cross-linking. The first step involved the fabrication of Gel/PLLA-MgO (GP@MgO) micro/nanofibrous mat (NF mat) [26,29]. The electrospun solution was prepared by dissolving 800 mg of gelatin, 200 mg of PLLA, and 50 mg of MgO NPs in 10 mL of HFIP. The prepared solution was stirred overnight at 20 °C and loaded into a 10 mL syringe for electrospinning. The solution was successively pumped at a flow rate of 3 mL/h using a syringe pump (Foshan Nanofiberlabs, Foshan, China) and electrospun at a potential of 17–19 kV between the spinneret (22 Gauge needle) using a high-voltage generator (Dongwen High Voltage, Tianjin, China). A ground mandrel with a rotating speed of 300 rpm was used to collect the nanofibers. The distance between the needle and the collector was 15 cm. The prepared GP-Mg mat was vacuum-dried to remove trace solvents. Subsequently, a short-fiber GP@MgO suspension was obtained in a tert-butanol solution using a grinder (Shanghai Jingxin, Shanghai, China). The sheared fiber mats were homogenized in tert-butanol at 2 % w/v with 25 Hz for 10 min at 25 °C. The homogenized suspension was transferred to 24-well plates, allowed to stand for 15 min at 25 °C, and stored in a −80 refrigerator overnight. The surface of the short fibers in direct contact with the well plate was considered to be the bottom surface of the scaffold, and the surface in contact with the air was the top surface. After thorough drying for 24 h using a freeze dryer (Foring, Beijing, China), the samples were crosslinked at 180 °C for 2 h in an oven to stabilize the structure. The GP aerogel was synthesized using the steps described above. The crosslinked aerogels were treated under vacuum for 24 h, and UV double-sided irradiation was performed for 6 h before *in vitro* cell culture and *in vivo* animal studies.

### 2.3 Characterization

Scanning electron microscopy (SEM, TESCAN MIRA LMS, Czech Republic) was used to observe aerogel morphology. The average fiber diameter and porosity were measured using the ImageJ software. The aerogels were weighed using an electronic balance (accuracy 0.0001 g), and their diameters and heights were measured using Vernier calipers (accuracy 0.02 mm). Aerogel volume and density were calculated using the following equations:

$$V=\pi r^{2}h$$

$$\rho \;\left(g.{\text{cm}}^{-3}\right)=m/V$$

where *ρ* is the density, and V, r, h, and m, refer to the aerogel's volume, radius, height, and mass, respectively.

Fourier-transform infrared spectroscopy (FTIR, Thermo Scientific iN10, USA) and X-ray diffraction (XRD, Bruker D8 Advance, Germany) were used to characterize GP, GP@MgO, and MgO NPs. A water contact angle analyzer (WCA, Dingsheng JY-82C, China) was used to measure the water contact angles of the GP and GP@MgO aerogels.

A universal testing machine (MTS CMT6103, USA) was used to test the GP and GP@MgO aerogels using compression tests. The aerogel was cylindrical with a height of 3 mm and a diameter of 10 mm. The change in stress at the degree of strain was recorded, and Young's modulus was calculated.

Inductively coupled plasma optical emission spectrometry (ICP–OES; Agilent 5110, USA) was used to quantify the Mg ions released from the GP@MgO aerogel. The aerogel was placed in 5 mL double-distilled water at 37 °C, and 1 mL liquid was taken at each time point for testing. To better align with clinical practice, a 0.1 M sodium acetate (Macklin, China) solution was prepared. The pH was adjusted to 6.0 using acetic acid (Macklin) and sodium hydroxide (Sinopharm, China), followed by supplementation with glucose (Sinopharm) to a final concentration of 20 mM. The specific procedures prior to testing were processed as per in double-distilled water.

Laser scanning microscopy (Keyence VK-X1000, Japan) was used to detect the different morphologies and roughness on each side of the aerogel, and a multi-file analyzer was used for 3D reconstruction.

### 2.4 Cell culture

Human umbilical vein endothelial cells (HUVECs) and 208F cells were cultured in Dulbecco's modified Eagle medium (DMEM). Bone marrow-derived mononuclear macrophages (BMDMs) were extracted as described previously [30] and cultured in minimum essential medium (MEM) containing recombinant rat macrophage colony-stimulating factor (M-CSF; PeproTech, USA). The medium was supplemented with 10 % fetal bovine serum (FBS) and 1 % penicillin-streptomycin and changed every 2 days. Furthermore, 100 ng/mL of lipopolysaccharide (LPS; Sigma) was added to the medium to mimic the inflammatory environment and aerogel was placed after the cell seeding plate with one per well floating on the medium in all *in vitro* experiments.

### 2.5 Cell biocompatibility and proliferation analysis

HUVECs or 208Fs were seeded in 12-well plates at a 2.5 × 10^4^ cells/well density and co-cultured for 1, 4, and 7 days with one aerogel per well. The cells were incubated with live-dead working solution (YEASEN Biotech, China, 2 μM Calcein AM, 4.5 μM PI) for 15 min and observed under the fluorescence microscope. In addition, the original medium was replaced with 10 μM 5-ethynyl-2-deoxyuridine (EDU, APE BIO, K1175, K1075, USA) working solution and incubated with HUVECs for 2 h and 208Fs for 4 h in 37 °C. After fixation and permeabilization, the cells were click-reacted and stained using 5 μg/mL Hoechst 33342 as per instructions. The plates were washed thrice with 3 % BSA (Sinopharm) in PBS (Servicebio, China) between steps.

HUVECs and 208Fs were seeded in 12-well plates at a density of 5 × 10^4^ cells/well for 3 days. After fixing 4 % PFA (Biosharp, China) for 15 min and blocking in 10 % goat serum for 2 h at 20 °C, the cells were incubated overnight at 4 °C with VEGF (1:200, affinity) and Col III (1:200, affinity). The cells were then incubated with fluorescein isothiocyanate-conjugated goat anti-rabbit IgG (H + L) [1:1000, APE BIO] and Cy3-conjugated goat anti-rabbit IgG (H + L) [1:1000, APE BIO] for 2 h. The nuclei were stained with 4’,6-diamidino-2-phenylindole (DAPI, 10 μg/mL, Biosharp) for 10 min. Images were obtained under various excitation conditions using a fluorescence microscope (Zeiss Axio Observer Z1, Germany).

### 2.6 Scratch and tube formation assay

HUVECs and 208Fs were seeded in 6-well plates at a density of 1.5 × 10^5^ for 24 h at 37 °C. A 200 μL tip was used to make parallel scratches on the bottom of the plate. The medium was changed to 2 % FBS to reduce the effects caused by cell proliferation. The scratch migration area was observed at 0, 12, and 24 h and photographed using a fluorescence microscope.

Matrigel diluent (100 μL) was spread evenly on pre-cooled 24-well plates and solidified at 37 °C for 30 min. 5 × 10^4^ HUVECs were seeded and incubated for 4 h. The cells were stained with Calcein AM for 10 min, and tube formation was observed using fluorescence microscopy.

### 2.7 Cellular senescence and mitochondrial function

HUVECs and 208Fs were seeded in 12-well plates at 5 × 10^4^ cells/well density for 3 days. Cell supernatants were taken for the enzyme-linked immunosorbent assay (ELISA) to detect SASP indicators (TNF-α, IL 1β, IL 6 and CCL2). The cells were stained with senescence-associated β-galactosidase (SA-β-gal) staining kit (Beyotime, China). First, the cells were fixed for 15 min using β-galactosidase fixative and then incubated overnight at 37 °C with the β-galactosidase working solution. Then, the samples were washed with 70 % ethanol to eliminate crystals and subsequently photographed. Besides, the plates were fixed, blocked, and stained with p21 (1:200; affinity), p16 (1:200, affinity) and γ-H2A.X (Beyotime) before being photographed under a fluorescence microscope.

HUVECs and 208Fs were seeded in 12-well plates at 5 × 10^4^ cells/well density for 3 days. The cells were incubated with mitochondrial membrane potential working solution (Solarbio, China) or 2′,7′-Dichlorodihydrofluorescein diacetate (DCFH-DA) working solution (Solarbio) for 20 min at 37 °C and photographed using fluorescence microscopy.

### 2.8 Macrophage phenotype

Briefly, cells derived from rat bone marrow were cultured in 20 ng/mL M-CSF complete medium for 24 h. The supernatant was transferred to a new cell culture bottle and cultured for 3 d. The BMDMs were seeded in 12-well plates and co-cultured with GP and GP@MgO aerogel for 24 h at 37 °C. After fixation and confinement, the cells were incubated overnight at 4 °C with CD86 (1:200, Proteintech), CD206 (1:200, Proteintech), TNF-α (1:200, affinity), or IL-10 (1:200, affinity), respectively. The F-actin was stained with rhodamine-labeled phalloidin (1:200, Solarbio) and FTIC-labeled phalloidin (1:200, Solarbio) for 30 min each. Nuclei were stained using DAPI for 10 min. Images were obtained at various fluorescence excitation wavelengths using a fluorescence microscope. Cells co-cultured with the aerogel for 24 h were collected and subjected to flow cytometry to detect macrophage polarization (BD Biosciences, Franklin Lakes, NJ, USA). The samples were incubated on ice in the dark for 30 min using the antibodies CD86 (1μg/test, Invitrogen, USA) and CD206 (1μg/test, Bioss).

Likewise, the BMDM cells were co-cultured with the aerogel for 12h. Samples from the cells were subjected to RT-PCR to quantify the mRNA expression levels of inducible nitric oxide synthase (iNOS), arginase-1 (Arg1), TNF-α and IL-10, and the cell culture supernatant was analyzed using an ELISA assay (TNF-α and IL-10). Total RNA was extracted from BMDMs using TRIzol reagent (Invitrogen). The concentration of RNA was determined using Nano Drop (Thermo Fisher Scientific, OH). Subsequently, gene expression analysis was conducted using the SYBR Green Premix pro Tag HS qPCR Kit (ACCURATE BIOLOGY, China) on a QuantStudio™ 5 Dx Real-Time PCR (Thermo Fisher Scientific). Relative gene expression was analyzed by the ΔΔCT method and the sequences of primers were presented in the Supplementary Material Table S1.

The BMDM cells in serum-free medium were seeded in the upper chamber of a Transwell plate (8.0 μm pore size, Corning, 3422), and medium with different aerogels were added to the lower chamber. After 12 h of co-culture, the cells were fixed and stained with crystal violet. The cells attached to the upper surface were removed and those on the lower surface were photographed. After the BMDM cells were co-cultured with aerogel for 24h, Mg^2+^ probe working solution (Mag-520 a.m., 5 μM, Maokang Bio) was replaced and incubated for 45 min, subsequently photographed.

### 2.9 Animal studies

All protocols for *in vivo* experiments were performed according to the NIH Guide for the Care and Use of Laboratory Animals. The Bengbu Medical University Animal Study Committee approved all procedures involved in animal experiments (Approval No. 2024602). Male Sprague–Dawley (SD) rats (200–240 g) were purchased from the Zhejiang Experimental Animal Center (Hangzhou, China). After fasting for 12 h, the rats were injected intraperitoneally with 1 % streptozotocin (STZ, 55 mg/kg, Solarbio). After 1 week of feeding, the STZ-injected rats had random blood glucose levels of 16.7 mM, with weight loss, polydipsia, and polyuria. These were considered successful models of type I diabetes and were used in subsequent experiments. Diabetic rats were randomly divided into three groups: Control, GP, and GP@MgO. After anesthesia, three 1 × 1 cm circular full-thickness skin defects were excised from the backs of the rats along the midline of the spine. Wound healing was recorded on days 0, 3, 7, 14, and 21, and samples were collected at these time points. The wound size was calculated using the following equation:

$$Wound\;size\;\left(\%\right)=S_{t}\;/S_{t}S_{0}\times 100\%$$

*S*_*0*_ is the wound area on day 0, and *S*_*t*_ is the wound area on days 3, 7, 14, and 21.

On day 21, rat serum samples and organs, including the heart, liver, spleen, lung, kidney, and pancreas, were collected to verify biosafety and type I diabetes.

### 2.10 Histology

The separated skin and organ tissues were soaked in 4 % PFA for 24 h, dehydrated with an ethanol gradient, embedded in paraffin, and sliced into 7 μm sections. Hematoxylin & eosin (HE) and Masson's trichrome staining assessed diabetic wound healing. The p21 (1:200, affinity) was used to evaluate the aging process. Platelet endothelial cell adhesion molecule-1 (CD31, 1:200, Proteintech) and alpha-smooth muscle actin (α-SMA, 1:100, affinity) were used as vascular markers to calculate the number of neovessels and assess the degree of revascularization. Collagen I (COL I, 1:100, affinity) and collagen III (COL III, 1:100, affinity) were used to evaluate collagen deposition. Cytokeratin-14 (CK14, 1:200, Proteintech) was used as an epithelial marker to assess the degree of re-epithelialization. iNOS (1:200, Proteintech) and Arg-1 (1:400, Proteintech) were used as macrophage markers to evaluate macrophage polarization. IL-1β and IL-4 were used as inflammatory factors to assess the level of inflammation. Insulin (1:400, Servicebio) and glucagon (1:400, Servicebio) were used as islet A and B cell markers, respectively, to evaluate the establishment of diabetes models.

### 2.11 Statistical analysis

All data are shown as the mean ± standard deviation. Statistical analyses were performed using Student's t-test and analysis of variance using GraphPad Prism (version 8.0.1). ImageJ was used to obtain measurements from the images. GraphPad Prism and Origin Pro (version 8.5) were used to plot the graphs. The significance levels were set as follows: ns (not significant, *p* > 0.05), ∗ (*p* < 0.05), ∗∗(*p* < 0.01), and ∗∗∗ (*p* < 0.001).

## 3 Results and discussion

### 3.1 Morphology and characterization of GP@MgO aerogel

**Fig. 1:**
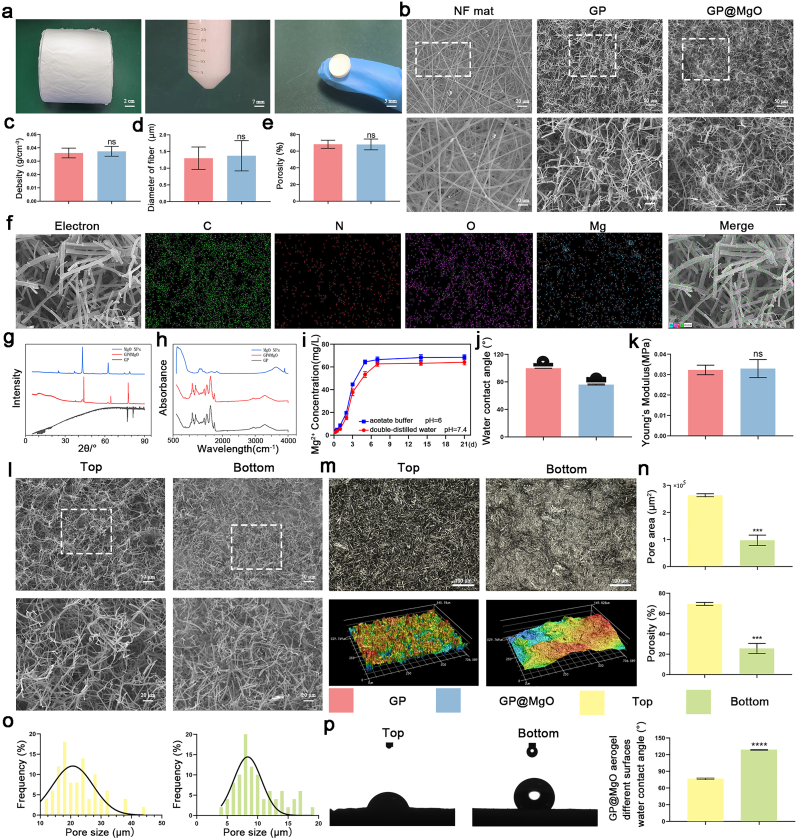
Morphology and characterization of GP@MgO aerogel. **a** Digital diagram of the key steps in aerogel preparation. **b** SEM images of NF mat, GP aerogel, and GP@MgO aerogel. **c** Aerogels density analysis. **d** Fiber diameter analysis. **e** Aerogels porosity analysis. **f** EDS elemental mapping of GP@MgO aerogel. **g** XRD patterns. **h** FTIR spectra. **i** Release of magnesium ions from GP@MgO aerogel. **j** Water contact angle. **k** Young's modulus. **l** SEM images of different surfaces of the aerogel. **m** Original image and 3D reconstructed image from laser scanning microscopy. **n** Pore aera and porosity of different surfaces of the aerogel. **o** Histogram of frequency distribution of different surfaces of the aerogel pore size. **p** Water contact angle and analysis of different surfaces of the aerogel. (ns, no significance and ∗∗∗∗*P* < 0.0001).

**Fig. 2:**
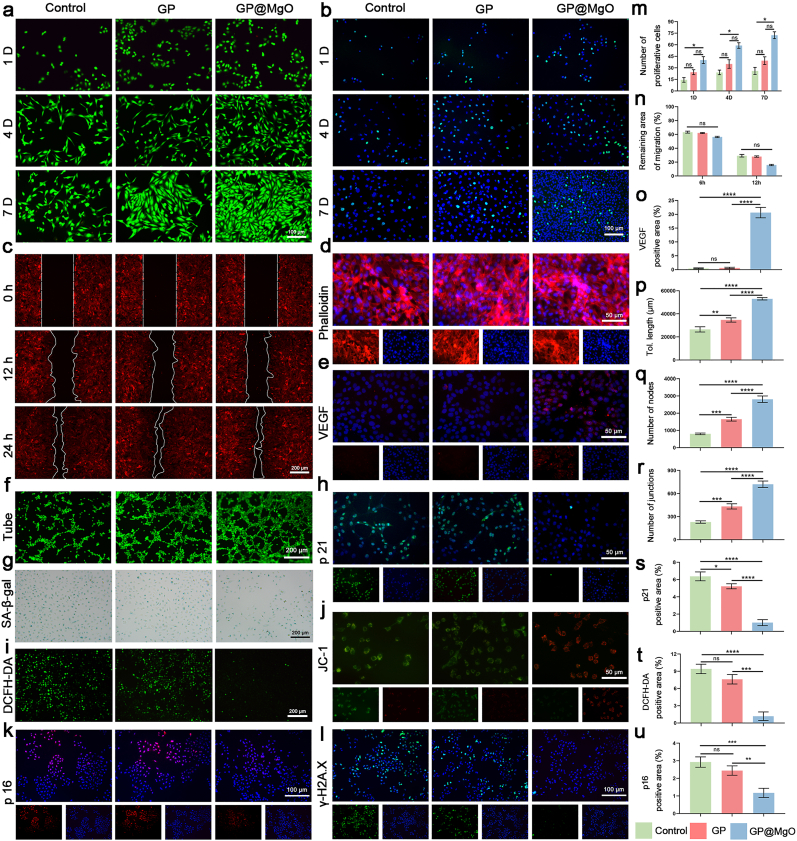
GP@MgO aerogels have excellent biocompatibility, promote cellular function, and mitigate senescence of endothelial cells. **a** Live/dead staining on days 1, 4, and 7 (scale bar: 100 μm). **b** EDU staining on days 1, 4, and 7 (scale bar: 100 μm) and **m** statistical analysis. **c** Cell migration (scale bar: 200 μm) and **n** statistical analysis. **d** Cytoskeleton staining (scale bar: 50 μm). **e** Immunofluorescence staining of VEGF (scale bar: 50 μm) and **o** statistical analysis. **f** tube formation (scale bar: 200 μm) and **p-r** statistical analysis of angiogenesis images. **g** SA-β-gal staining (scale bar: 200 μm). **h** Immunofluorescence staining of p21 (scale bar: 50 μm) and **s** statistical analysis. **i** DCFH-DA staining (scale bar: 200 μm) and **t** statistical analysis. **j** JC-1 staining (scale bar: 50 μm). **k** Immunofluorescence staining of p16 (scale bar: 100 μm) and **u** statistical analysis. **l** Immunofluorescence staining of γ-H2A.X (scale bar: 100 μm). (ns, no significance; ∗*P* < 0.05, ∗∗*P* < 0.01, ∗∗∗*P* < 0.001, and ∗∗∗∗*P* < 0.0001).

The relevant characterization of the MgO NPs is shown in Supplementary Fig. S1. The SEM images indicated that the particle size was approximately 40 nm, and energy-dispersive X-ray spectroscopy (EDS) suggested the presence of overlapping Mg and oxygen elements. GP@MgO short fiber aerogels with ECM structures were prepared via electrospinning, pulverization, freeze-drying, and cross-linking. The two-dimensional micro/nanofibrous mat was prepared by electrospinning a Gel/PLLA/MgO NPs solution in HFIP. Then, the fiber mats were crushed in a tert-butanol solution. Finally, it was dried and crosslinked to stabilize the short fiber aerogel structure (Fig. 1a). The NF mats were tightly packed with overlapping layers, as evidenced by EDS images showing typical fiber layers and morphology (Fig. 2b and Fig. S2). The SEM data revealed that the upper surface of the GP and GP@MgO short fiber aerogels disrupted the regular network structure of the fiber mats, exhibiting irregular and folded micro/nanofiber with loose and porous structures (Fig. 2b). The short fiber aerogels had fiber diameters of approximately 1 μm, ultra-low density, and high porosity; there was no significant difference between GP and GP@MgO (Fig. 1c, d, and 1e). Meanwhile, EDS analysis indicated a distribution of MgO NPs in GP@MgO aerogels (Fig. 1f). Furthermore, the existence of MgO NPs in the fibers was further confirmed by XRD analysis (Fig. 1g). In the XRD standard pattern, the MgO NPs exhibited a sharp diffraction peak at 2θ = 42.82° [31]. The diffraction peaks observed for the self-synthesized MgO NPs and GP@MgO aerogel confirmed the successful preparation and loading of the NPs. The FTIR spectra of the MgO NPs, GP aerogel, and GP@MgO aerogel are shown in Fig. 1h. Given the susceptibility of MgO NPs to water absorption and hydrolysis, which results in the formation of magnesium hydroxide, the absorption peak of the hydroxyl group at 3700 nm can be employed as a marker. However, this peak was not observed for the aerogel due to the relatively low concentration of MgO NPs [32]. The release of Mg ions is shown in Fig. 1i. The GP@MgO aerogels were placed in acidic acetate buffer and neutral double-distilled water, respectively. The release of Mg was rapid during the initial 3-day period, reaching a plateau on day 7 in double-distilled water. The release rate in acetate buffer was comparable to that of the double-distilled water group during the initial 3-day, likely due to the fibers' capacity to retain the internal MgO NPs to a certain degree. However, the peak time was reached within the day 5, earlier than the double-distilled water group. Furthermore, the water contact angle on the upper surface of the GP aerogel was higher than that of GP@MgO (Fig. 1j). The mechanical properties of the short fiber aerogels is shown in Fig. 1k. There was no notable discrepancy between GP and GP@MgO regarding Young's modulus.

Fig. 1l showed SEM images of different surfaces of the aerogel, exhibiting different fiber densities and porosities. The top fibers were sparse and porous, whereas the bottom fibers were dense and heavily stacked. The 3D reconstructed laser scanning microscope images showed the morphologies and roughness of the different surfaces of the aerogels (Fig. 1m). The top image displays numerous pores and substantial longitudinal extensions, while the bottom image exhibits the relatively flat shape of the short fibers after stacking downwards under gravity. There were significant differences in pore area, porosity and pore size distribution observed between different surfaces aerogels (Fig. 1n and o). Fig. 1p showed the hydrophilic and hydrophobic properties of the short fiber aerogels based on the water contact angle. Notably, there was a discrepancy in the degree of hydrophilicity between the top and bottom of the aerogels: the bottom fibers were flat, but the water contact angle exceeded 120°, while the upper surface of the aerogel was not uniform, and the water contact angle was less than 90°.

Biomaterial scaffolds should ideally mirror the natural extracellular matrix and adapt to the intricacies of localized defects to promote tissue regeneration. Conventional electrospun membranes are dense stacks of fibers with small pore sizes and low porosity, which are not conducive to cell growth and are, therefore, not ideal for use in biomedical applications [33]. Short fiber aerogels exhibit a three-dimensional porous structure by remodeling the structure of crushed fibers [26]. The short fibers are initially subjected to physical entanglement, enhancing the mechanical bonding. Meanwhile, the substantial number of amino groups in gelatin and the carboxyl groups in PLLA facilitate the formation of peptide bonds through thermal cross-linking, further enhancing the mechanical properties and stability [34]. Furthermore, the differing hydrophilicity observed on the two sides of the samples can be attributed to the uneven fiber density resulting from the gravitational settling of the short fibers within the tertiary butanol solution following crushing and dispensing. The resulting variations in fiber density affect the surface tension and pore size, which in turn affect the Laplace pressure and the adhesive tension [35,36]. The rate of liquid absorption is directly proportional to the capillary radius, and spreads rapidly upon contact with the hydrophilic layer [37,38]. The liquid is drawn in by capillary force and gradually absorbed by the aerogels to maintain a dry environment for the wound bed. The exposed portion evaporates the absorbed liquid and increases the liquid absorption ratio [39]. Therefore, the GP@MgO short fiber aerogel can absorb and evaporate extravasated tissue fluid from the wound bed over time while gradually exposing the loaded MgO NPs.

### 3.2 Biocompatibility and of GP@MgO aerogel *in vitro*

The unique structure of aerogels composed of entangled nanofibers allows them to replicate natural ECM configurations, enabling them to regulate various cellular activities, including cell proliferation, migration, and secretion.

**Fig. 3:**
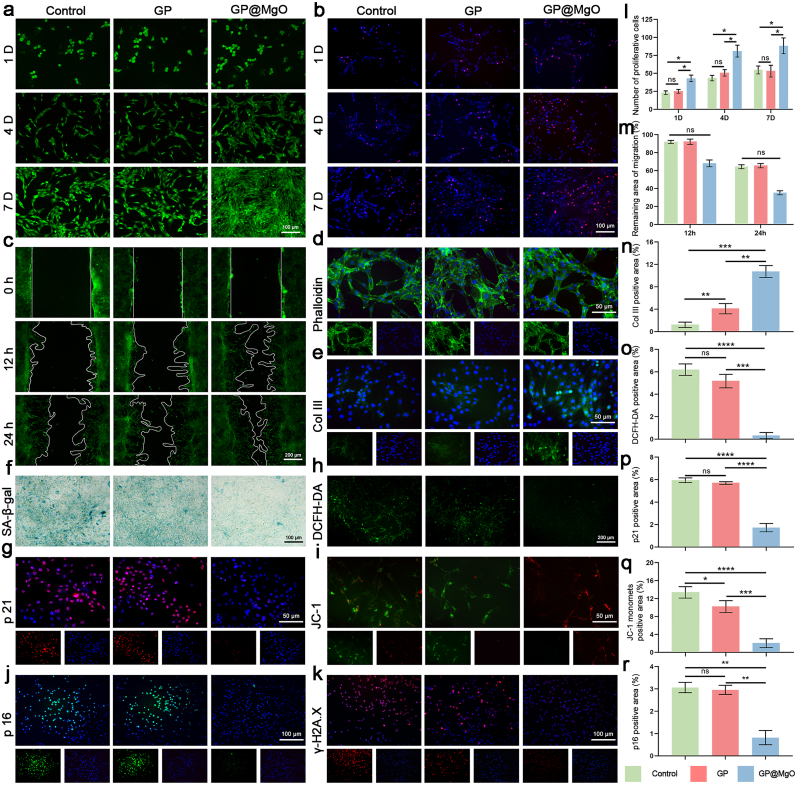
GP@MgO aerogels have excellent biocompatibility, promote cellular function, and mitigate the senescence of fibroblasts. **a** Live/dead staining on days 1, 4, and 7 (scale bar: 100 μm). **b** EDU staining on days 1, 4, and 7 (scale bar: 100 μm) and **l** statistical analysis. **c** Cell migration (scale bar: 200 μm) and **m** statistical analysis. **d** Cytoskeleton staining (scale bar: 50 μm). **e** Immunofluorescence staining of Col Ⅲ (scale bar: 50 μm) and **n** statistical analysis. **f** SA-β-gal staining (scale bar: 100 μm). **g** Immunofluorescence staining of p21 (scale bar: 50 μm) and **p** statistical analysis. **h** DCFH-DA staining (scale bar: 200 μm) and **o** Statistical analysis. **i** JC-1 staining (scale bar: 50 μm) and **q** statistical analysis. **j** Immunofluorescence staining of p16 (scale bar: 100 μm) and **r** statistical analysis. **k** Immunofluorescence staining of γ-H2A.X (scale bar: 100 μm). (ns, no significance; ∗*P* < 0.05, ∗∗*P* < 0.01, ∗∗∗*P* < 0.001, and ∗∗∗∗*P* < 0.0001).

As shown in Fig. 2, Fig. 3a, the cell number increased over time, and the cell morphology was stretched, particularly in the GP@MgO group. Live/dead cell staining revealed an almost negligible number of dead cells. The number of proliferating cells was assessed using EDU staining on days 1, 4, and 7. GP@MgO exhibited a higher fluorescence level than the control and GP groups, indicating the promotion of cell proliferation (Fig. 2, Fig. 3b and l). The impact of the GP@MgO aerogel scaffolds on cell migration was evaluated by scratching (Fig. 2, Fig. 3c and m). The GP@MgO exhibited a higher cell migration rate to the damaged area than the other groups at 12 and 24 h. Nuclear skeleton staining showed a fully expanded cytoskeleton, indicating that the aerogel scaffolds were not cytotoxic (Fig. 2, Fig. 3d). Furthermore, GP@MgO significantly potentiated VEGF expression (Fig. 2e and o). A comparison of the different groups of nodes and tube lengths treated with different aerogels for 4 h revealed that the formation of GP@MgO was significant, with the control group showing almost no tube formation (Fig. 2f, p, 2q, and 2r). In addition, GP@MgO upregulated the expression of Col III (Fig. 3e and n). In conclusion, the GP@MgO aerogel enhanced the physiological activity of HUVEC and 208F cells and offered the possibility of *in vivo* neovascularization and collagen deposition.

### 3.3 Retardation of cellular senescence and modulation of macrophage polarization of GP@MgO aerogel *in vitro*

Microenvironmental imbalances in patients with diabetes caused by fluctuations in blood glucose levels create adverse conditions for oxidative stress, collagen synthesis, angiogenesis, cell proliferation, and other tissue regenerative processes [40]. The sustained elevation of glucose in an organism results in an acidic shift within the surrounding microenvironment, prompting the release of factors belonging to the pro-inflammatory factor family and the deformation of the extracellular matrix [41]. This continued inflammatory state damages mitochondrial function and delays ROS clearance [9]. Meanwhile, the inflammatory response at the traumatic defect site further intensifies the already hostile microenvironment, thereby contributing to immune-induced senescence [4,15]. Therefore, it is evident that the high-glucose environment associated with diabetes renders organisms incapable of effectively addressing the consequences of external trauma.

We investigated the senescence phenotypes of HUVECs and 208Fs to address this issue. Cells exhibited increased SA-β-Gal activity under LPS-mimicked inflammatory induction (Fig. 2, Fig. 3f). However, adding the GP aerogel did not alleviate this phenomenon. In contrast, GP@MgO resulted in a notable downregulation of expression. Similarly, the GP@MgO aerogel demonstrated a substantial decrease in the expression of senescence biomarker p21 and p16 (Fig. 2h, s, 2k and 2u, Fig. 3g, p, 3j and 3r). Furthermore, the expression of the DNA damage marker γ-H2A.X was similarly inhibited by GP@MgO aerogel (Fig. 2, Fig. 3k). The relevant SASP indexes, as detected by ELISA, also indicated significant suppression of expression in the GP@MgO group (Fig. S3 and S4). Research on mitochondrial function has provided further insights into the causes of cellular senescence. Based on DCFH-DA (Fig. 2, Fig. 3h and o), ROS fluorescence expression was suppressed and significantly lower than in the other groups. Examination of mitochondrial membrane potential yielded consistent findings (Fig. 2, Fig. 3i). JC-1 staining showed significant depolarization of the mitochondrial membrane potential following immune induction, accompanied by a notable increase in JC-1 monomer levels. However, repolarization was observed after GP@MgO treatment, indicating the recovery of inflammation-induced mitochondrial function.

**Fig. 4:**
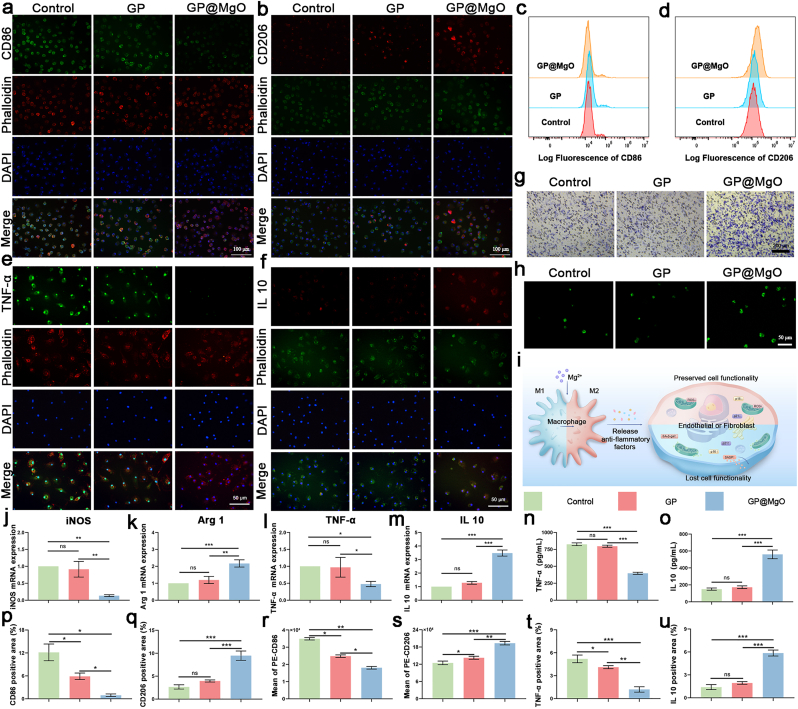
GP@MgO aerogels mediate the macrophage phenotype and SASP expression. **a** Immunofluorescence staining of CD86 (scale bar: 100 μm) and **p** statistical analysis. **b** Immunofluorescence staining of CD206 (scale bar: 100 μm) and **q** statistical analysis. **c** Flow cytometry histograms of CD86 and **r** quantitative analysis. **d** Flow cytometry histograms of CD206 and **s** quantitative analysis. **e** Immunofluorescence staining of TNF-α (scale bar: 50 μm) and **t** statistical analysis. **f** Immunofluorescence staining of IL 10 (scale bar: 50 μm) and **u** statistical analysis. **g** Transwell migration experiment (scale bar: 200 μm). **h** Probe staining for Mg^2+^ (scale bar: 50 μm). **i** Schematic illustration of senescence inhibition by Mg^2+^. **j-m** RT-PCR results showing the levels of iNOS, TNF-α, Arg 1 and IL 10. **n-o** Levels of TNF-α and IL 10 in supernatants of BMDM cells by ELISA. (ns, no significance; ∗*P* < 0.05, ∗∗*P* < 0.01 and ∗∗∗*P* < 0.001).

**Fig. 5:**
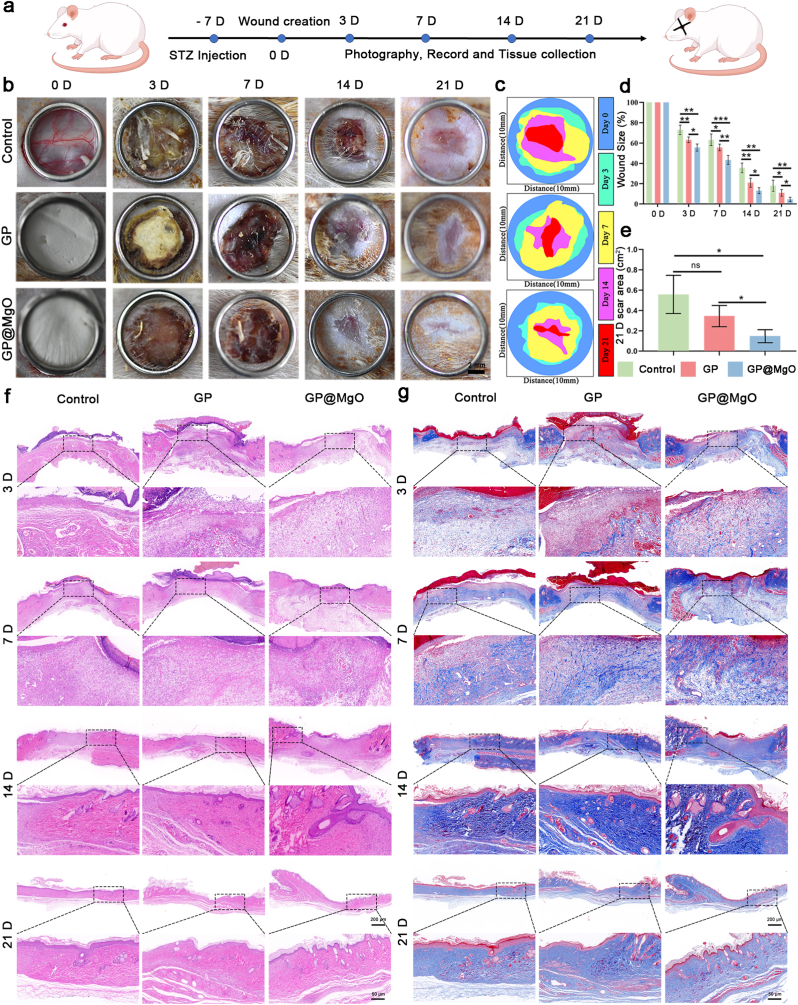
GP@MgO aerogels accelerate wound healing in diabetic rats. **a** Schematic illustration of *in vitro* experiment. **b** Representative wound closure images in diabetic rats at each time point. **c** Wound area digital reconstruction. **d** Percentage of the wound area. **e** Wound area on day 21. **f** H&E staining (scale bar: 200 μm and 50 μm). **g** Masson's trichrome staining (scale bar: 200 μm and 50 μm). (ns, no significance; ∗*P* < 0.05, ∗∗*P* < 0.01 and ∗∗∗*P* < 0.001).

In organismal immunity, we further validated the modulation of immunity by the GP@MgO aerogel through its effects on macrophage polarization and inflammatory cytokine secretion. Immunofluorescence staining was completed by labeling M1 and M2 cell surface markers (CD86 and CD206) after inducing BMDM differentiation (Fig. 4a, b, 4p, and 4q). The LPS-stimulated control group showed an increased proportion of M1 macrophages, whereas the coculture of M1 macrophages with the GP aerogel remained highly expressed. However, GP@MgO treatment reduced the number of M1 macrophages and increased the expression of M2 markers. Moreover, comparing the results using flow cytometry revealed a consistent trend with the fluorescence expression, with significantly stronger CD206 expression observed in GP@MgO than in the other groups (Fig. 4c, d, 4r, and 4s). Furthermore, as shown in Fig. 4e and f, consistent outcomes were demonstrated by comparing the M2-representative marker IL-10 and SASP representative marker TNF-α. To further validate the regulatory effects of GP@MgO aerogel on the inflammatory environment, the expression levels of different phenotypic marker genes (*iNOS*, Arg *1*, *TNF-α* and *IL 10*) of macrophages were investigated at the mRNA level through RT-PCR (Fig. 5j–m). Inflammatory indicators from the ELISA (TNF-α and IL 10) also provided consistent validation (Fig. 5n and o). GP@MgO treatment led to a notable elevation in the expression level of anti-inflammatory factors. It considerably impeded the secretion of SASP compared with the control and GP groups. Meanwhile, according to the transwell migration assay, showed that the GP@MgO group demonstrated a notablenotably enhancement in cell counts and had a substantial impact on macrophage recruitment (Fig. 5g). Mg^2+^ probe staining revealed high fluorescence intensity following treatment with GP@MgO (Fig. 5h), indicating that macrophages absorb pre-hydrolyzed Mg^2+^ and enhance function. From the above results, it was inferred that GP@MgO significantly enhanced macrophage polarization towards M2 in an inflammatory environment and improved the hostile microenvironment to retard immune-induced senescence.

The released MgO NPs from the aerogel react with water to form magnesium hydroxide (Mg(OH)_2_), which has partial solubility in water and fully disintegrates into Mg^2+^ and hydroxide ions (OH^−^). The high-glucose microenvironment in diabetes causes chronic inflammation, which results in inflammation and accelerates the aging process in organisms [42]. Dissociated OH^−^ effectively neutralizes hydrogen ions (H^+^) in the acidic diabetic microenvironment and lactic acid formed by glycolysis in post-traumatic hypoxia, which adjusts the pH of the local microenvironment [43,44]. It has been previously shown that an increase in pH within the microenvironment can effectively downregulate aging markers (p16, p21) through the PI3K/AKT/mTOR pathway [17]. The continuous depletion of the OH^−^ level results in a persistent hydrolysis reaction of MgO, which maintains the release of Mg^2+^. On the one hand, Mg^2+^ has been shown to safeguard mitochondrial functionality by mitigating the decline in mitochondrial membrane potential [45]. Similarly, the instability of mitochondrial membrane potential functions as an early indicator of apoptosis and the stabilization of the potential helps to reduce cell death [46,47]. The process of hydrolysis of MgO NPs in a diabetic environment is pivotal to maintaining mitochondrial function and restoring the imbalanced energy metabolism, which in turn preserves cellular function and accelerates means of tissue repair.

Furthermore, the intricate interplay between metal ions and the immune system is pervasive in innate and adaptive immunity [14]. Macrophages are the first to be recruited in large numbers to the wound site following damage, and they considerably influence immune modulation [48]. Magnesium ions have been used as precursors for a range of enzymatic reactions and have been shown to display a wide range of biological activities [49]. Research has demonstrated that magnesium ions have the capacity to induce macrophage polarization towards M2 and exert regulatory effects through the NF-κB pathway [50,51]. To elaborate, Mg^2+^ inhibits the translocation of p65 from the perinuclear to the intranuclear area by curbing the activation and phosphorylation of IκB [52]. Similarly, Mg^2+^ can slow aging by inhibiting the inward flow of calcium ions, regulating the SIRT1-p53 pathway, and reducing SASP secretion [17]. On the other hand, Mg^2+^ modulates the expression of downstream inflammatory factors (IL-1β and IL-10) through a dual pathway, thereby affecting inflammation. A harsh inflammatory environment impairs mitochondrial function, leading to increased levels of oxidative stress, cellular senescence, and the secretion of SASP factors [53]. By reducing harmful transmission signals downward from external immune cells, improves the survival environment of endothelial and fibroblast cells. Due to the reduction of external stimuli, the vicious cycle between “ROS accumulation - cellular senescence” is interrupted, thus effectively preserving the function of damaged cells and saving the surrounding unaffected or minimally damaged cells.

### 3.4 Evaluation of diabetic wound healing

Based on *in vitro* results showing that the GP@MgO aerogel regulated cell proliferation, angiogenesis, and macrophage polarization, a diabetic full-thickness wound model was established to validate further the aerogel's effect on diabetic wound healing *in vivo*. In the type I diabetes model, random blood glucose values in diabetic rats were consistently >16.7 mmol/L during the healing period, accompanied by a significant decrease in body weight during the early stages (Fig. S5a and S5b). Pancreatic islets are shown in Fig. S5c.

A digital camera (Canon, Japan) was used to record the wound healing process, as shown in Fig. 5b. The degree of healing varied in each group exposed to the same feeding environment during the treatment period. The wound area after treatment with the GP@MgO aerogel was smaller than that after treatment with the control and GP aerogels at each time point, and the healing process was notably faster. The untreated wound was observed to be moist with a visible yellow exudate on day 3; on day 7, it was incompletely crusted and still exuded. The GP-and GP@MgO-treated wound beds were observed to be dry, and yellow exudates were adsorbed onto the GP aerogel surface on day 3. The scab completely covered the wound on day 7. The wounds treated with GP@MgO on day 21 exhibited a high degree of healing as the treatment progressed. In contrast, the control group still has a large wound area. The dynamic healing of each group throughout the healing process was digitally reconstructed (Fig. 5c) to visualize changes in the wounds. The quantitative results at each time point (Fig. 5d and e) showed that the residual wound area of GP@MgO was less than that of the GP and control groups, indicating a positive effect of MgO NPs on diabetic wound healing.

HE staining was performed to evaluate further the *in vivo* healing results (Fig. 5f). The trauma length gradually shortened with healing time in each group. The thickness of granulation tissue gradually increased with time. The control and GP groups exhibited minimal neoplastic tissue, whereas the GP@MgO group demonstrated a notable abundance of granulation tissue on day 7. However, the GP@MgO group showed a decrease in thickness on day 21 compared to previous measurements. The result showed that the wounds in the GP@MgO aerogel group achieved re-epithelialization and initiated granulation tissue remodeling, whereas those in the control and GP groups exhibited delayed wound healing. Moreover, more complex epidermal structures, including skin appendages, were observed in the GP@MgO group on day 21 compared with those in the other groups. Furthermore, regeneration of hair follicles in each group was followed by Masson staining (Fig. 5g). No intact hair follicles were formed in the control group on day 14. In contrast, the GP@MgO group exhibited mature and numerous follicles. The discrepancy in the number of hair follicles among the groups on day 21 was even more pronounced. These results indicated that the GP@MgO aerogel can significantly accelerate the diabetic healing process and shorten healing time. The *in vivo* biosafety of GP and GP@MgO was proven by sectioning rats' hearts, livers, spleens, lungs, and kidneys, with no significant damage to the major organs (Fig. S6).

### 3.5 Cellular senescence and re-epithelialization *in vivo*

**Fig. 6:**
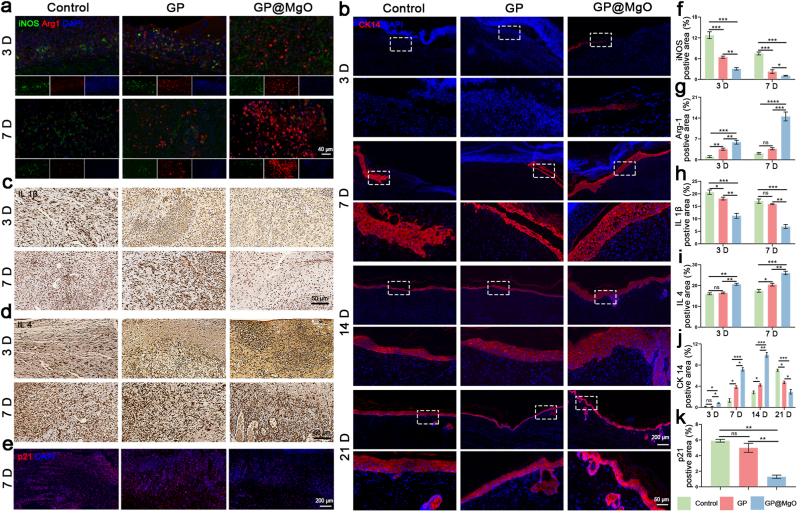
GP@MgO aerogels decrease inflammation, mitigate senescence and promote re-epithelialization. **a** Immunofluorescence staining of iNOS (green) and Arg 1 (red) and **f-g** quantitative analysis (scale bar: 40 μm). **b** Immunofluorescence staining of CK 14 (red) and **j** quantitative analysis (scale bar: 200 μm and 50 μm). **c** Immunohistochemical staining of IL 1β and **h** quantitative analysis (scale bar: 50 μm). **d** Immunohistochemical staining of IL 4 and **i** quantitative analysis (scale bar: 50 μm). **e** Immunofluorescence staining of p21 (red) and **k** quantitative analysis (scale bar: 200 μm). (∗*P* < 0.05, ∗∗*P* < 0.01, ∗∗∗*P* < 0.001, and ∗∗∗∗*P* < 0.0001). (For interpretation of the references to colour in this figure legend, the reader is referred to the Web version of this article.)

The optimal approach for managing diabetic wounds is to regulate the progression of the inflammatory phase. Macrophages are pivotal in the inflammatory phase, act as vital indicators for initiating regeneration, and are key predictors of healing outcomes [54]. Macrophage immunofluorescence in all groups on days 3 and 7 were stained with iNOS and Arg-1 (Fig. 6a). At the outset of the inflammatory process (day 3), the control group exhibited significantly elevated iNOS levels compared with those of the GP@MgO group, indicating heightened inflammatory activity. However, the GP@MgO group had a significant population of M2 macrophages at both time points, suggesting that MgO NPs facilitated the transition of macrophages from M1 to M2. Notably, GP aerogel did not significantly reduce the number of M1 macrophages. Conversely, it increased the expression of Arg-1, which ultimately allowed the wound to progress from the inflammatory phase into the proliferative phase (Fig. 6f and g). In light of the above, we further evaluated the intensity of the inflammatory response through immunohistochemical analysis of IL 1β and IL 4 (Fig. 6b, h, and 6c, 6i). The highest expression of IL-1, a pro-inflammatory factor marker, was observed on day 7 in the control group, while the lowest expression was noted in the GP@MgO group. Similarly, the GP@MgO group exhibited significantly higher expression levels of IL-4, an anti-inflammatory marker, than the control and GP aerogel groups. These results demonstrate that the GP@MgO aerogel is an effective modulator of the inflammatory phase in diabetic wounds. Therefore, to further validate the effect of controlled inflammation on wound tissue senescence, p21 staining was performed to assess the degree of senescence (Fig. 6e and k). Compared with the control group, the positive area of p21 was significantly reduced after treatment with GP@MgO, indicating successful mitigation of the senescence marker p21. Collectively, a combination of *ex vivo* and *in vivo* experiments demonstrated that the GP@MgO aerogel short fiber scaffolds could ameliorate the immune microenvironment in diabetes and rescue inflammation-mediated senescence.

Cytokeratin (CK) is an important biomarker associated with epidermal differentiation and re-epithelialization [55]. CK14, an intermediate fibrin type I keratin family member, is expressed during undifferentiated complex epithelial development and forms basal keratin in composite squamous epithelial keratin cells when paired with the type II keratin CK5 [56,57]. Immunofluorescence staining for CK14 showed that the expression of red-fluorescent CK14 was strong at each time point in the new epidermis of the GP@MgO group (Fig. 6b). Meanwhile, the epithelial thickness on day 21 was lower than that on day 14 in the GP@MgO group. This was accompanied by the appearance of several hair follicles, indicating that the wound had entered the remodeling phase. In contrast, the epithelial thickness continued to increase in the control group. These results showed that wound tissue re-epithelialization was accelerated, and healing characteristics were enhanced in the GP@MgO group compared with those in the control and GP groups (Fig. 6j). These findings were consistent with those of the HE and Masson staining.

### 3.6 Collagen deposition and neovascularization *in vivo*

**Fig. 7:**
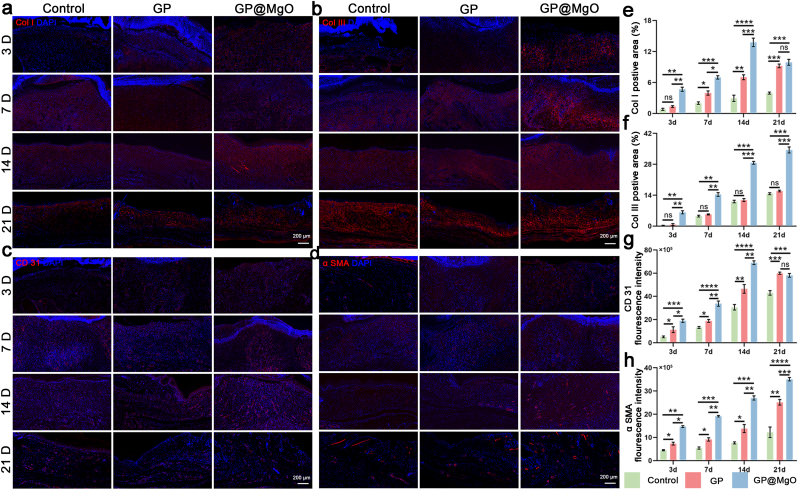
GP@MgO aerogels accelerate collagen deposition and neovascularization. **a** Immunofluorescence staining of Col I (red) and **e** quantitative analysis (scale bar: 200 μm). **b** Immunofluorescence staining of Col III (red) and **f** quantitative analysis (scale bar: 200 μm). **c** Immunofluorescence staining of CD 31 (red) and **g** quantitative analysis (scale bar: 200 μm). **d** Immunofluorescence staining of α SMA (red) and **h** quantitative analysis (scale bar: 200 μm). (∗*P* < 0.05, ∗∗*P* < 0.01, ∗∗∗*P* < 0.001, and ∗∗∗∗*P* < 0.0001). (For interpretation of the references to colour in this figure legend, the reader is referred to the Web version of this article.)

The orderly deposition of collagen contributed to ECM restructuring of the extracellular matrix and wound healing [58]. The deposition and remodeling of Col I and Col III were identified through immunofluorescence at various healing time points following different scaffold treatments (Fig. 7a and b, Fig. S7a and S7b). Sporadic collagen formation was observed only in the GP@MgO group on day 3. Thereafter, there was a notable increase in the expression levels across all groups. However, the GP@MgO group significantly outperformed the other groups. A balanced arrangement of mature bundled collagen fibers was observed, particularly on days 14 and 21. Overall, the expression of Col III was higher than that of Col I (Fig. 7e and f).

The insufficient neovascularization typical of diabetic wounds represents a significant obstacle to the normal healing process [59]. Immunofluorescence staining was employed to observe angiogenesis, with CD31 and α-SMA employed to label neovascularization and mature vessels, respectively. As shown in Fig. 7c and Fig. S8a, the control group did not exhibit any new blood vessel formation on day 3, whereas the GP@MgO group demonstrated neovascularization. On day 7, neoplastic microvessels were observed in all the groups. The GP@MgO group had a significantly superior outcome regarding the number of vessels and tube size. On day 14, both the GP and GP@MgO groups exhibited the emergence of small and medium-sized vessels. On day 21, the newborn capillaries in the GP@MgO group degenerated, with only medium-sized vessels remaining. By contrast, the control group showed capillaries and small vessels, indicating that the trauma had not yet entered the remodeling phase. Although α-SMA levels were observed to be simultaneously lower than those of CD31, the intergroup trend was consistent with that of CD31 (Fig. 7d and h, Fig. S8b). In particular, the blood vessel diameters labeled by CD31 and α-SMA in the GP@MgO group on day 21 were highly similar, and the absence of capillaries further corroborated that the wounds had entered the remodeling phase. These findings indicated that the GP@MgO-treated wounds followed the proper wound repair pathway in a time-dependent manner.

This study had some limitations. Although we demonstrated that GP@MgO short-fiber aerogels can control the inflammatory microenvironment by modulating macrophages to reduce SASP secretion and slow local aging, the mechanism by which specific molecules regulate immune-induced aging remains unclear. Therefore, future studies should aim to investigate the role of proteins and nucleic acids in the biological activity of skin composition-associated cells during this process.

## 4 Conclusions

In this study, a biomimetic three-dimensional MgO NPs-loaded aerogel (GP@MgO aerogel) was prepared via electrospinning, pulverization, freeze-drying, and crosslinking to accelerate diabetic wound healing and hinder immune-induced senescence. The GP@MgO aerogel kept the wounds dry by effectively absorbing tissue exudates through its three-dimensional porous structure. This mitigates the prognosis of inflammatory senescence by modulating the macrophage phenotype and improving the inflammatory microenvironment by releasing internal MgO NPs. Furthermore, the aerogels promoted angiogenesis, collagen deposition, and re-epithelialization, thus significantly accelerating diabetic wound healing. The benefits of the GP@MgO aerogel could provide a potential way to mitigate inflammatory aging in chronic wounds and a new treatment for diabetic wound closure.

## CRediT authorship contribution statement

**Yedan Chen:** Writing – original draft, Methodology, Investigation, Funding acquisition, Conceptualization. **Qingxiang Liu:** Validation, Methodology, Investigation, Formal analysis. **Jingjing Guan:** Validation, Resources, Methodology. **Chunyang Zheng:** Validation, Formal analysis. **Shumeng Shi:** Validation, Formal analysis. **Weiwei Zheng:** Writing – review & editing, Supervision, Conceptualization. **Jianzhong Guan:** Writing – review & editing, Supervision, Conceptualization. **Yingji Mao:** Writing – review & editing, Supervision, Project administration, Funding acquisition, Conceptualization.

## Declaration of competing interest

The authors declare that they have no known competing financial interests or personal relationships that could have appeared to influence the work reported in this paper.

## Data Availability

Data will be made available on request.
