# Supplementary material for: Rescuing inflammatory microenvironment induced senescence via MgO immune mediated asymmetric short fibers for accelerating diabetic wound repair
Source: Mater Today Bio. 2025 Jul 13;33:102079. doi: 10.1016/j.mtbio.2025.102079 (PMC12284504; doi:10.1016/j.mtbio.2025.102079)
Supplement: Multimedia component 1 [file mmc1.docx]

**Supplementary Material:**

**Rescuing inflammatory microenvironment-induced senescence via MgO immune-mediated** **asymmetric short fibers for accelerating diabetic wound repair**

Yedan Chen^a,b,1^, Qingxiang Liu^a,1^, Jingjing Guan^a,1^, Chunyang Zheng^b,c^, Shumeng Shi^b,c^,Weiwei Zheng^d,*^, Jianzhong Guan^a,*^, Yingji Mao^a,b,c,*^

^a^ Department of Orthopedics, Department of Plastic Surgery, and Department of Rehabilitation Medicine, The First Affiliated Hospital of Bengbu Medical University, Bengbu 233004, China

^b^ Anhui Nerve Regeneration Technology and Medical New Materials Engineering Research Center, School of Life Sciences, Bengbu Medical University, Bengbu 233030, China

^c^ Anhui Provincial Key Laboratory of Tumor Evolution and Intelligent Diagnosis and Treatment, Bengbu Medical University, Bengbu 233030, China

^d^ Department of Orthopedics, The Affiliated Suzhou Hospital of Nanjing Medical University, Gusu School, Nanjing Medical University, Suzhou 215000, China

^1^ Yedan Chen, Qingxiang Liu, and Jingjing Guan contributed equally to this work.

^*^ Corresponding Authors:

Weiwei Zheng, Department of Orthopedics, The Affiliated Suzhou Hospital of Nanjing Medical University, Gusu School, Nanjing Medical University, Suzhou 215000, China; Email: zhengweiweidoctor@163.com.

Jianzhong Guan, Department of Orthopedics, The First Affiliated Hospital of Bengbu Medical University, Bengbu 233004, China; Email: guanjianzhong@bbmc.edu.cn.

Yingji Mao, Anhui Nerve Regeneration Technology and Medical New Materials Engineering Research Center, School of Life Sciences, Bengbu Medical University, Bengbu 233030, Anhui, China; Email: myj123@bbmu.edu.cn


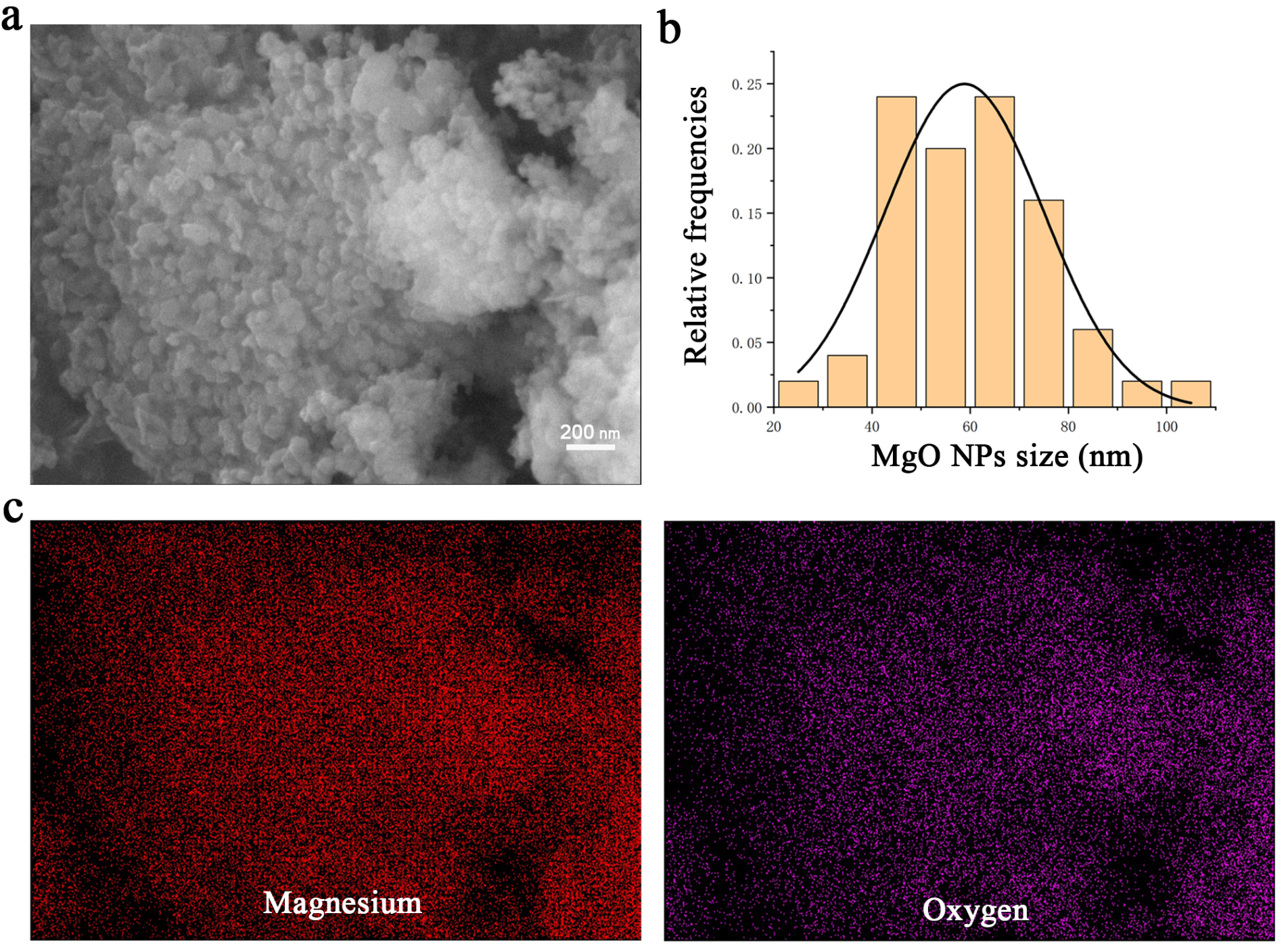


**Fig S1:** MgO nanoparticle morphology. **a** Representative SEM image. **b** Nanoparticle size distribution. **c** EDS elemental mapping of Mg (red) and O (purple).


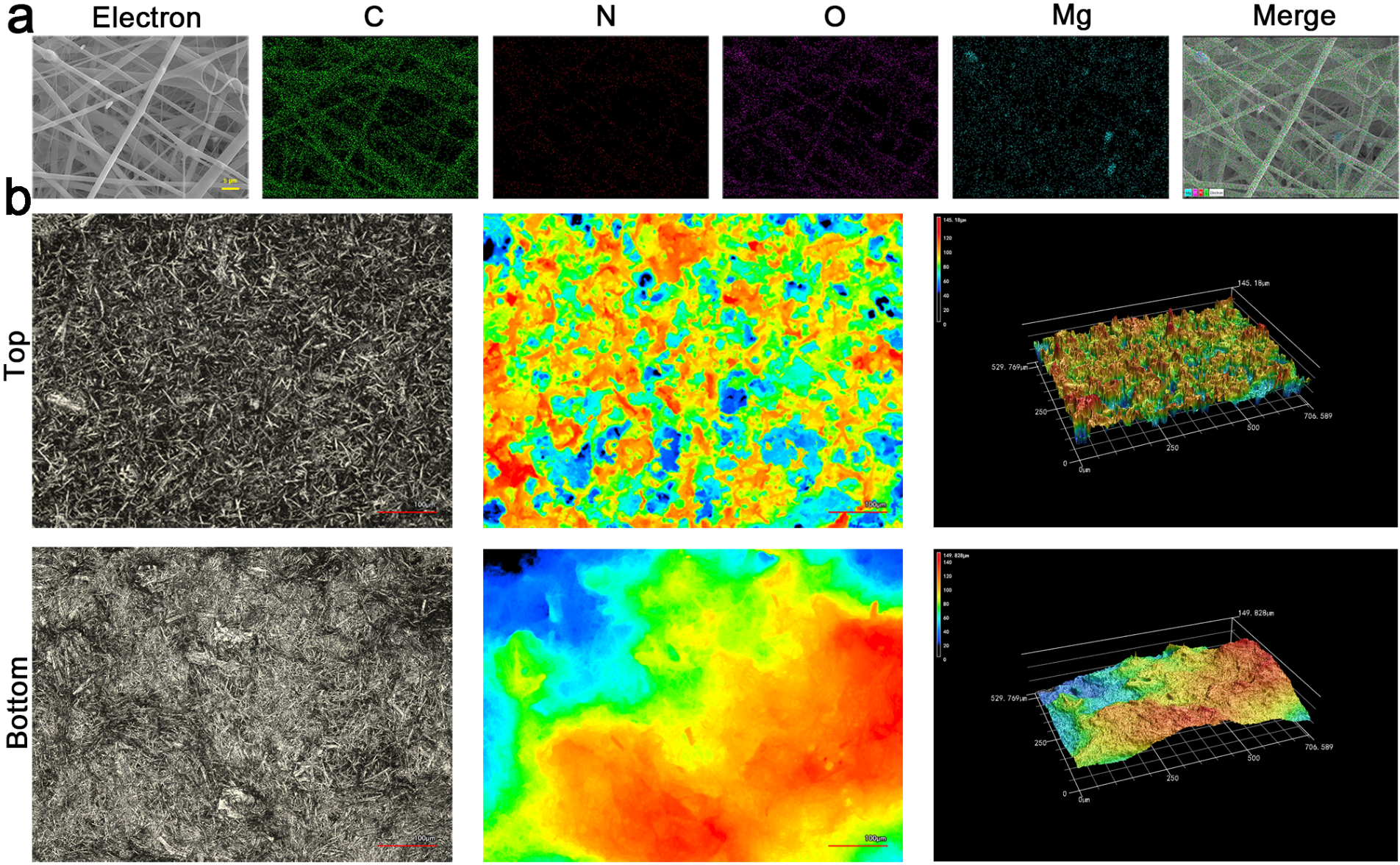


**Fig S2:** Characterization. **a** Elemental composition and image combination of micro/nanofiber mats. **b** Laser scanning microscopy electron and 3D reconstructed images.


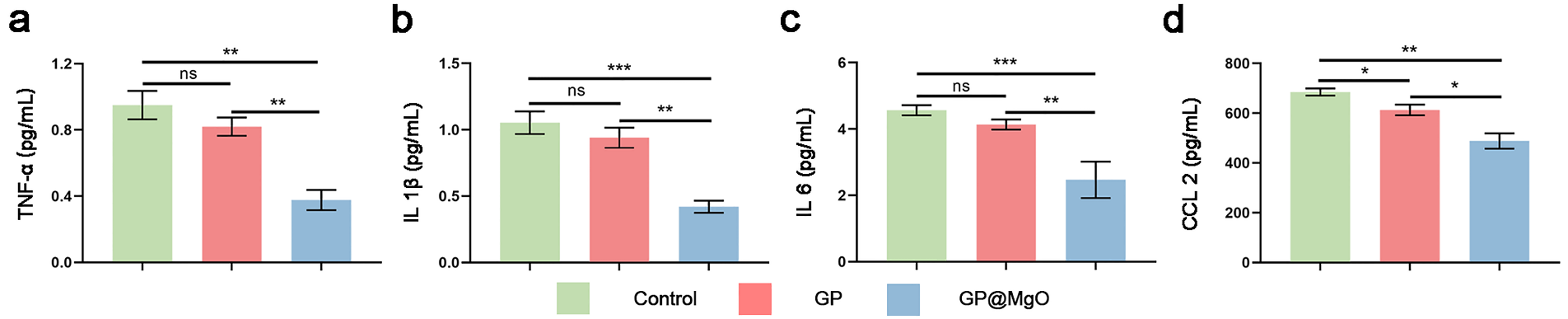


**Fig S3:** Levels of SASP indicators in HUVEC cells supernatants by ELISA. **a** TNF-α. **b** IL 1β. **c** IL 6. **d** CCL2. (ns, no significance; **P* < 0.05, ***P* < 0.01 and ****P* < 0.001)


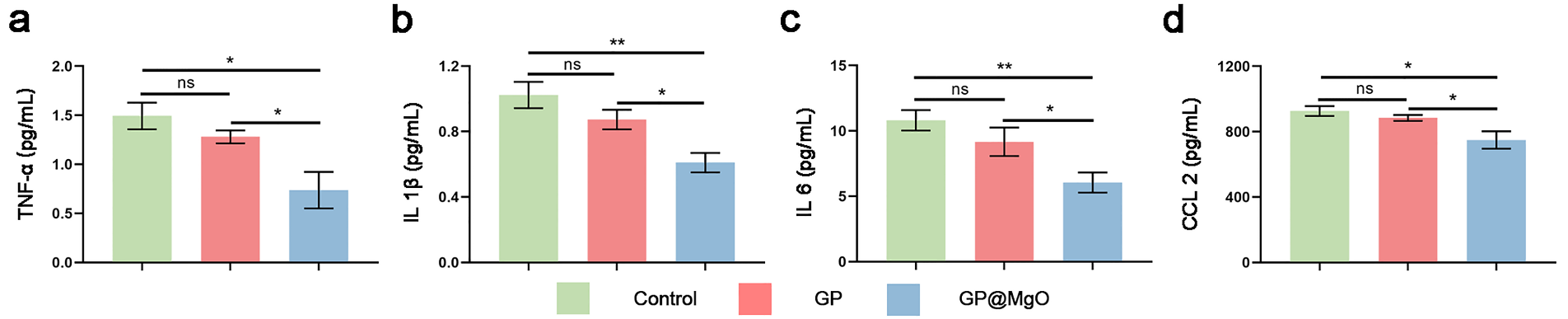


**Fig S4:** Levels of SASP indicators in 208F cells supernatants by ELISA. **a** TNF-α. **b** IL 1β. **c** IL 6. **d** CCL2. (ns, no significance; **P* < 0.05 and ***P* < 0.01)


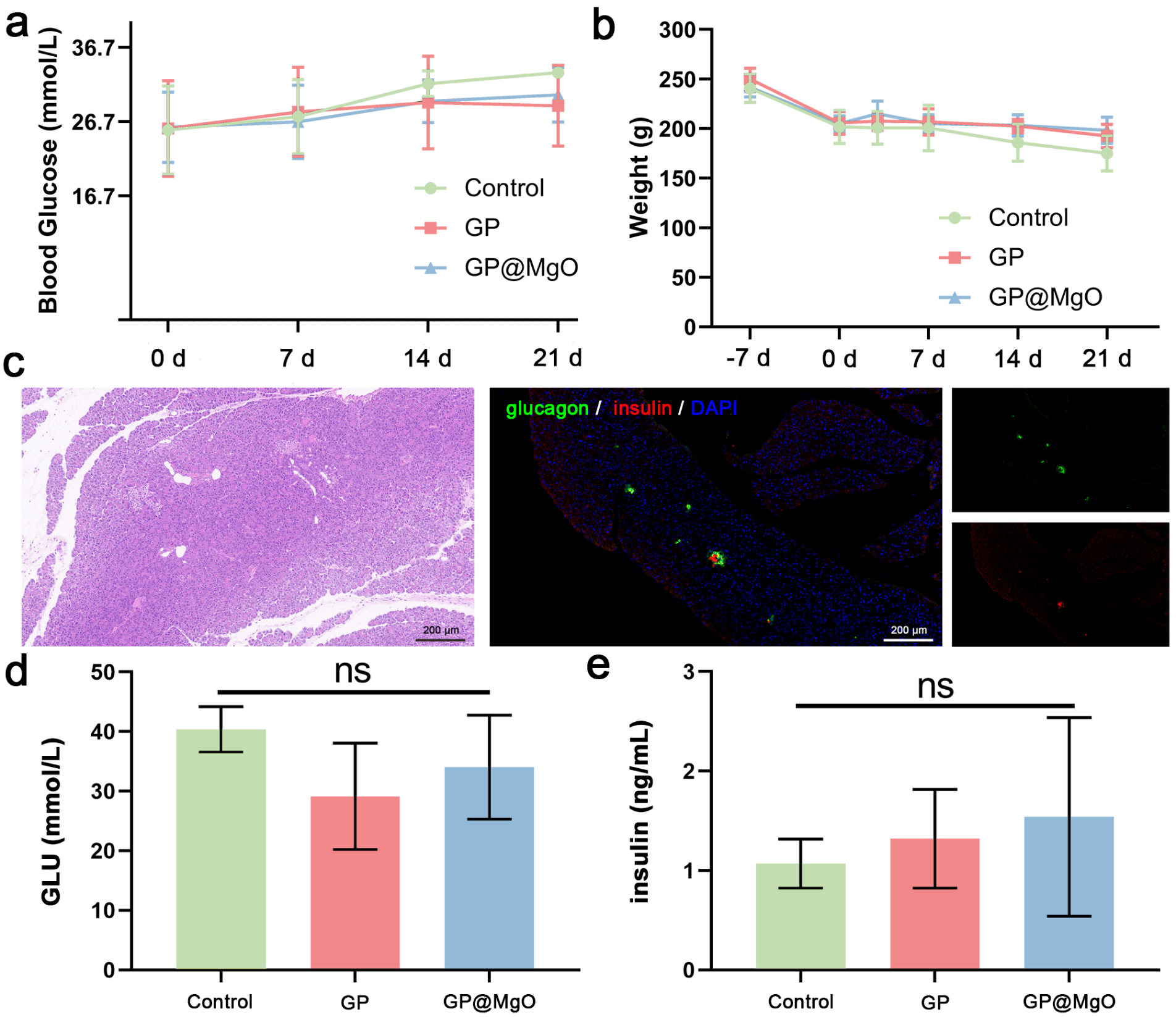


**Fig S5:** Identification and presentation of a rat model of type 1 diabetes. **a** Rat blood glucose shift curves (glucometer measurement). **b** Rat weight transformation curves. **c** Representative HE and fluorescence staining of the pancreas of type Ⅰ diabetic rats. **d** ELISA for glucose. **e** ELISA for insulin. (ns, no significance)


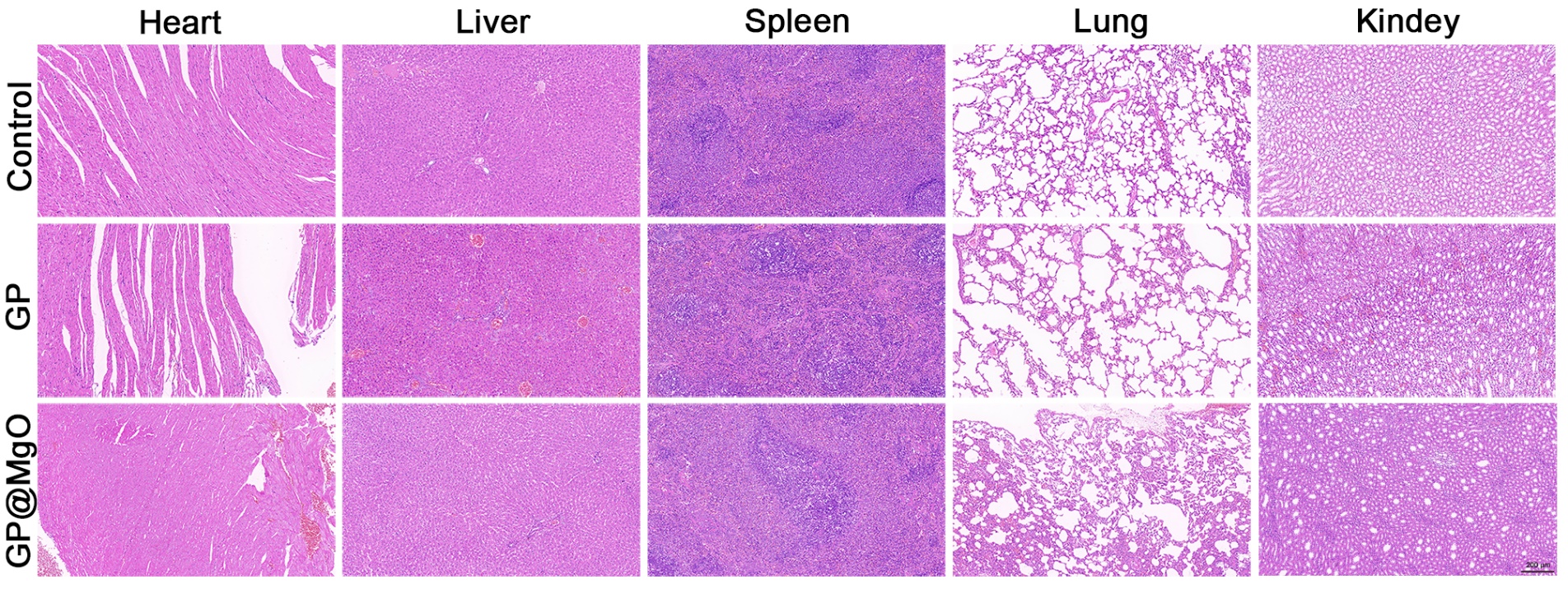


**Fig S6:** The biosafety testing of aerogels such as HE staining of critical organs, including heart, liver, spleen, lung, and kidney (scale bar: 200 μm).


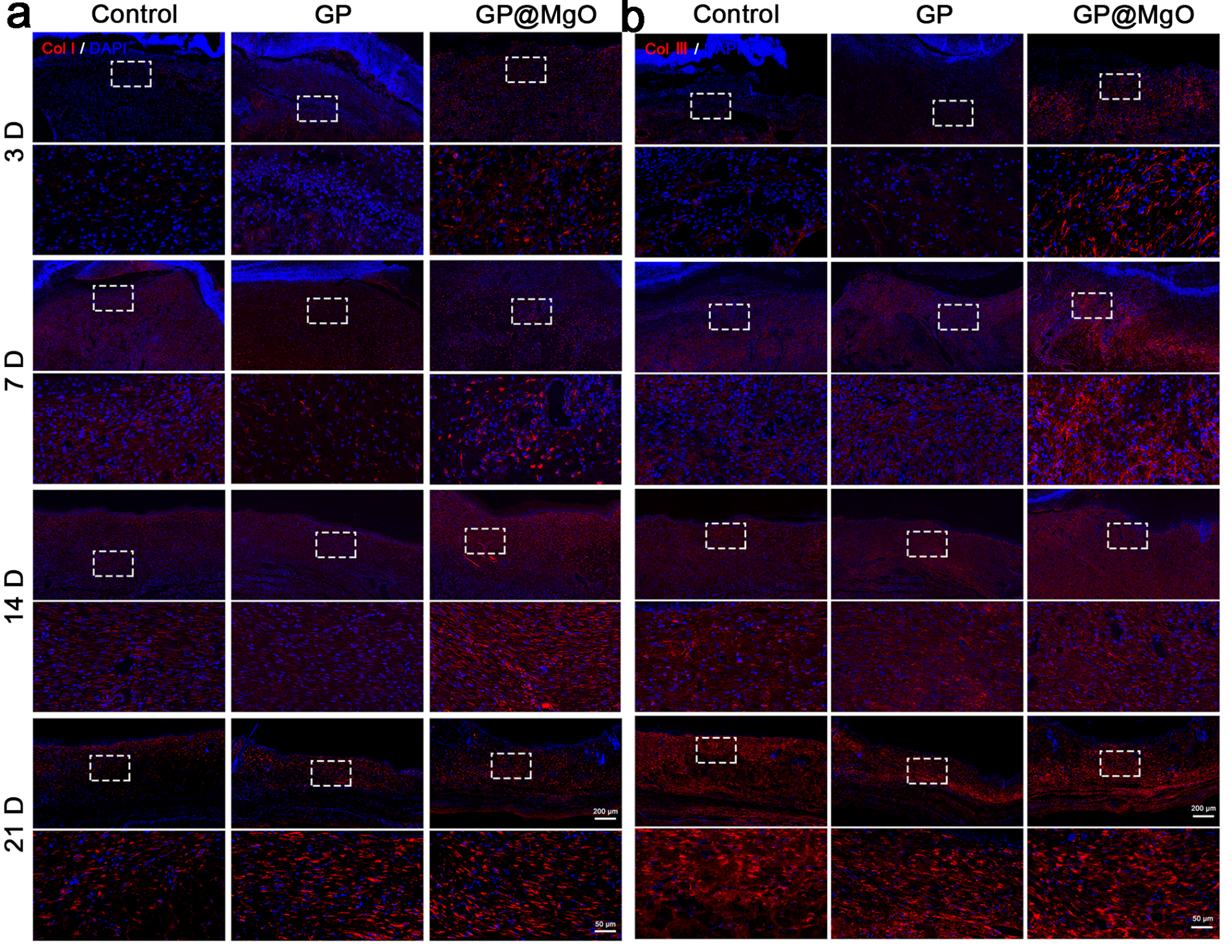


**Fig S7:** GP@MgO aerogel enhanced collagen neogenesis and ordered deposition in vivo. **a** Immunofluorescence staining of Col I (red, scale bar: 200 μm and 50 μm). **b** Immunofluorescence staining of Col III (red, scale bar: 200 μm and 50 μm).


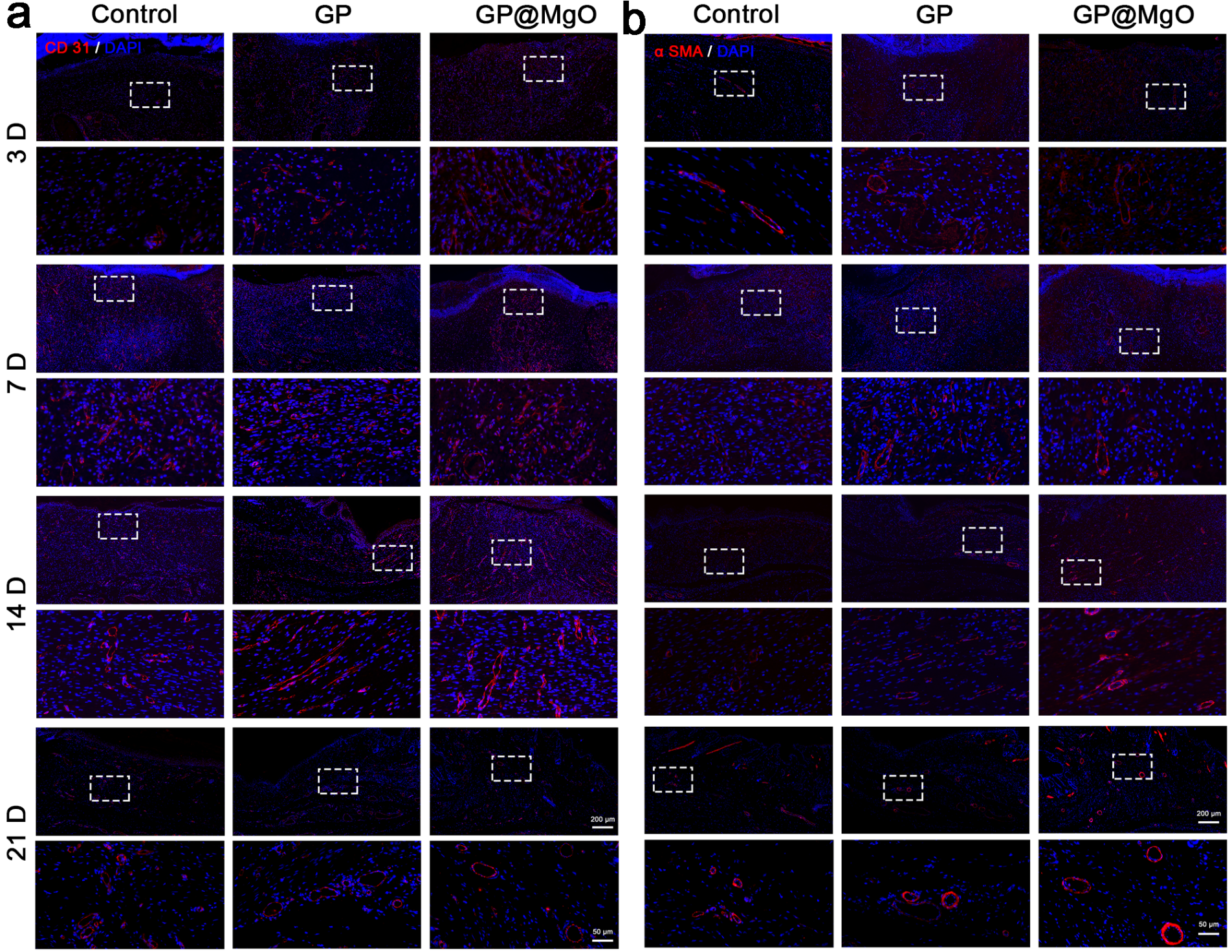


**Fig S8:** GP@MgO aerogel accelerated neovascularization in vivo. **a** Immunofluorescence staining of CD 31 (red, scale bar: 200 μm and 50 μm). **b** Immunofluorescence staining of α-SMA (red, scale bar: 200 μm and 50 μm).

Table S1

| **Gene** | **Forward primer** | **Reverse primer** |
| --- | --- | --- |
| Rat iNOS | TCACCTTCGAGGGCAGCCGA | CAGACGCCATGGTGCAGGGG |
| Rat Arg 1 | ATTCACCCCGGCTACGGGCA | AGGAGCAGCGTTGGCCTGGT |
| Rat TNF α | GCAGATGGGCTGTACCTTATC | GAAATGGCAAATCGGCTGAC |
| Rat IL 10 | AGTGGAGCAGGTGAAGAATG | GAGTGTCACGTAGGCTTCTA |
| Rat GAPDH | GGCCAAGGTCATCCATGA | TCAGTGTAGCCCAGGATG |
